# Development on Light and Thin Broadband Sound Absorption Structure Based on Unequal-Cross-Section Microperforated Plate Series Connection

**DOI:** 10.3390/ma17246282

**Published:** 2024-12-22

**Authors:** Xin Jv, Jinwu Wu, Qibo Mao, Qi Li, Tianhang Zhang

**Affiliations:** School of Power and Energy, Nanchang HangKong University, 696 South Fenghe Avenue, Nanchang 330063, China; 13123839770@163.com (X.J.); qbmao@nchu.edu.cn (Q.M.); l921123187@163.com (Q.L.); tianhang151600@163.com (T.Z.)

**Keywords:** microperforated plate, broadband sound absorption, unequal cross-section, series connection

## Abstract

The sound absorption structure of a microperforated plate has many advantages and has great potential in the field of noise control. In order to solve the problem of broadband sound absorption of microperforated plates, a series acoustic structure of microperforated plates of unequal cross-section was designed based on the traditional microperforated plate series acoustic structure. Compared with the traditional series structure, the sudden change of cross-section increases the sound energy dissipation and greatly improves the sound absorption performance. Through the analysis of its parameters, when the overall thickness of the structure is 20 mm, its sound absorption coefficient is above 0.5 in the frequency range of 1000–3450 Hz; there are three formants, and the sound absorption coefficients corresponding to the three formants reach 1. This study provides new ideas and methods for the design of broadband acoustic structures.

## 1. Introduction

With the development and progress of society, problems caused by noise are increasingly emphasized, and noise control is an important measure to improve the living environment. Noise can be effectively reduced by means of sound absorption, and microperforated plate (MPP) absorbers have been widely recognized and applied in noise control [1].The MPP structure and its detailed theory were first proposed by Maa in 1973 [2]. The structure utilizes the Helmholtz resonance cavity acoustic principle. When the sound wave is incident to the MPP, the friction with the hole turns the acoustic energy into thermal energy, which results in noise reduction. An MPP structure has the advantages of simple structure, easy production and low cost of materials, so it can be widely used in the field of acoustic absorption [3,4,5].

However, usually, the single-layer MPP acoustic structure has only one absorption peak, and its sound absorption range is narrow [6,7]. In recent years, many experts and scholars have conducted research on this basis to broaden the frequency range of sound absorption. Maa is also the first to propose a double-layer MPP structure to produce two resonance absorption peaks to broaden the absorption bandwidth [2]. On this basis, Bucciarelli proposed a seven-layer MPP structure to achieve a wider sound absorption bandwidth [8]. A multi-layer MPP acoustic structure with different back-cavity depths is proposed to increase the frequency range of acoustic absorption [9]. Gai proposed an L-shaped segmentation of the back cavity and combined it with an inhomogeneous MPP [10]. There are many scholars who have also carried out the same work as above, also through the MPP series or parallel structure, to realize multiple resonance, so that the generation of more resonance sound absorption peaks achieves the effect of broadband sound absorption [11,12,13,14,15,16,17,18]. The series and parallel connection of the MPP sound-absorbing structure is a way to achieve broadband sound absorption, and other scholars have achieved the goal by designing the structure of MPP combined with other materials. For example, MPP structures are combined with membranes [19,20], porous materials [21,22,23], shunt loudspeakers [24,25], Helmholtz resonators [26,27] and other materials to improve sound absorption performance. Zhang presents a new class of parallel-connected Helmholtz resonators with embedded apertures [28].

The design of acoustic structures with adjustable frequency of sound absorption has also been a hot research topic in the past few years. The sound absorption structure usually carries out passive sound absorption. Its structural parameters are determined, and the sound absorption frequency is relatively narrow, which has limitations in the face of complex and changeable noise environments. Yan designed a double-layer honeycomb microperforated structure with adjustable back-cavity height to modify the acoustic absorption performance [29]. Zhang incorporated a mechanical rotating mechanism into the cavity of the microperforated acoustic structure to change the perforation ratio of the microperforated plate to change the frequency of acoustic absorption [30]. Yang proposed an adjustable parallel Helmholtz resonator [31], which achieves band tunability through a guide rail and a slider. There are also some special films to achieve frequency band tunability. On the basis of the dielectric elastic film, a microporous dielectric elastomer can be formed [32,33], and the purpose of adjustable sound absorption can be achieved by changing the tension of the film and the size of the micropores by voltage.

The above are three main methods to achieve broadband sound absorption. However, these methods are mainly used to increase the overall thickness of the acoustic structure or to complicate the structure in order to improve the acoustic performance, which are methods difficult to apply to engineering practice.

In this paper, based on the traditional microperforated plate series acoustic structure, a microperforated plate with unequal cross-sections is proposed to be connected in series, to increase the sound speed and thus increase the loss of acoustic energy through the sudden change of the cross-section, and to produce multiple resonant acoustic peaks to realize the broadband acoustic absorption, which greatly improves the acoustic absorption performance and reduces the falloff between the peaks and valleys, compared to the traditional series connection with the same thickness. The sound absorption performance is greatly improved compared with the traditional series connection of the same thickness, and the drop between the peak and the valley is reduced.

In this paper, the proposed structure is first analyzed theoretically by the transfer matrix method, followed by parametric impact analysis of various parameters of the structure in Section 3. Then, simulation analysis is carried out in Section 4 and Section 5 to make predictions and experimental analysis to verify the reliability of the theory. Finally, the paper is summarized in Section 6.

## 2. Theoretical Analysis

**Figure 1:**
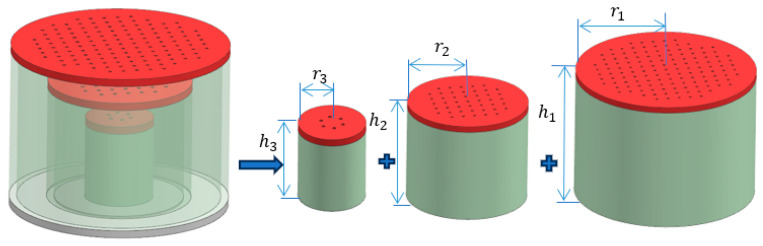
Microperforated plate series structure with unequal cross-section.

The sound absorption structure of the microperforated plate designed in this paper is shown in Figure 1, where $h_{i},r_{i}$ are the radius and the depth of the back cavity of the ith layer of the microperforated plate, respectively. It is a series structure of microperforated plates with unequal cross-sections, and the total size of the structure is a cylinder with a diameter of 29 mm and a height of 20 mm. The structure is light and thin, with a thickness of only 20 mm. Compared with the sound absorption of porous materials, the structure is composed only of microperforated plates with different sections [34], which is simple to make. It is based on the resonance between the sound waves and the structure to achieve the purpose of energy consumption.

**Figure 2:**
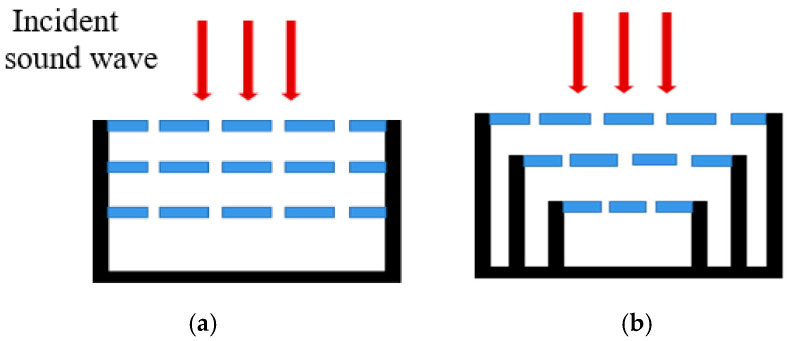
(**a**) Conventional multi-layer microperforated plate series structure; (**b**) Multi-layer micro-perforated plate series structure with unequal cross section.

There are two main methods for the theoretical calculation of the sound absorption structure of the microperforated plate, namely, the acoustoelectric analogy method and the transfer matrix method. For the structure with multi-layer microperforated plates connected in series, as shown in Figure 2, the acoustic impedance derived by the acoustoelectric analogy method has a large error from the actual due to the fact that the acoustic mass of its back cavity cannot be neglected. Zhang [30] compared the two methods, and it is clearly observed in the study that the acoustic absorption valleys are more obvious in the transfer matrix method considering the cavity acoustic mass. Therefore, the transfer matrix method is used to calculate the structure of multi-layer microperforated plates in series [35].

Firstly, according to the theoretical model of MPP proposed by Maa [2], the acoustic impedances of the single-layer microperforated plate can be obtained by the traditional microperforated plate theory:

(1) $$Z_{MPP}=\rho c\left(r+j\omega m\right)$$

Which are, respectively:

(2) $$r=\frac{0.147t}{pd^{2}}\left[\sqrt{1+\frac{k^{2}}{32}}+\frac{\sqrt{2}}{8}\frac{kd}{t}\right]$$

and

(3) $$m=\frac{0.294\times 10^{-3}}{p}\left[1+\sqrt{\frac{1}{9+\frac{k^{2}}{2}}}+0.85\frac{d}{t}\right]$$

The microperforated plate constant:

(4) $$k=d\sqrt{\frac{f_{0}}{10}}$$

In the above formulas, $Z_{MPP}$ is the relative acoustic impedance of the microperforated plate; ρ, c are the density of air and the speed of sound, respectively; t is the plate thickness; d is perforation diameter, in mm; p is the percentage of the total perforation area in the whole plate; and f is the sound frequency.

The transfer matrix calculation process of the traditional multi-layer microperforated plate series structure (taking three layers as an example) is as follows:

(5) $$T_{{MPP}_{i}}=\left[\begin{array}{cc} 1 & Z_{{MPP}_{i}}\\ 0 & 1 \end{array}\right]$$

(6) $$Z_{{MPP}_{i}}=\rho c\left(r_{i}+j\omega m_{i}\right)$$

(7) $$T_{D_{i}}=\left[\begin{array}{cc} \cos\left(\frac{\omega D_{i}}{c}\right) & j\rho csin\left(\frac{\omega D_{i}}{c}\right)\\ \frac{jsin\left(\frac{\omega D_{i}}{c}\right)}{\rho c} & \cos\left(\frac{\omega D_{i}}{c}\right) \end{array}\right]$$

Here, $Z_{{MPP}_{i}}$ is the acoustic impedance of the ith-layer microperforated plate, and $D_{i}$ is the back-cavity depth of the ith-layer microperforated plate. The overall transfer matrix of the three-layer microperforated plate in series is, then, as follows:

(8) $$T_{t}=\left[T_{{MPP}_{1}}\right]\cdot \left[T_{D_{1}}\right]\cdot \left[T_{{MPP}_{2}}\right]\cdot \left[T_{D_{2}}\right]\cdot \left[T_{{MPP}_{3}}\right]\cdot \left[T_{D_{3}}\right]$$

The total acoustic impedance of the structure can be calculated from the overall transfer matrix:

(9) $$Z_{t}=\frac{T_{t_{11}}}{{\rho cT}_{t_{21}}}$$

For the structure of unequal-cross-section microperforated plates in series designed in this paper, referring to the cross-section mutation acoustic structure designed by Yan [36], the cross-sectional areas of the upper and lower layers of the microperforated plates are also different, and since the mutation of the cross-section makes the speed of sound increase, a velocity-change matrix should be added.

(10) $$B_{i}=\left[\begin{array}{cc} 1 & 0\\ 0 & \frac{S_{i+1}}{S_{i}} \end{array}\right]$$

(11) $$S_{i}=\pi \cdot {r_{i}}^{2}$$

where $S_{i}$ is the area of the ith layer of the microperforated plate, and $r_{i}$ is the radius of the ith layer of the microperforated plate. The back-cavity depth *D* at this point is different from the back-cavity depth of the conventional structure, and the equivalent cavity depth is used here for the calculation:

(12) $$D_{1}=\left(S_{1}\cdot h_{1}-S_{2}\cdot h_{2}\right)/S_{1}$$

(13) $$D_{2}=\left(S_{2}\cdot h_{2}-S_{3}\cdot h_{3}\right)/S_{2},D_{3}=h_{3}$$

where $D_{i}$ is the equivalent back-cavity depth of the ith layer of the microperforated plate. Thus, the overall transfer matrix of the tandem structure of microperforated plates with unequal cross-sections can be obtained as follows:

(14) $$T_{t}=\left[T_{{MPP}_{1}}\right]\cdot \left[T_{D_{1}}\right]\cdot \left[B_{1}\right]\cdot \left[T_{{MPP}_{2}}\right]\cdot \left[T_{D_{2}}\right]\cdot \left[B_{2}\right]\cdot \left[T_{{MPP}_{3}}\right]\cdot \left[T_{D_{3}}\right]$$

Similarly, according to Equation (9), the total acoustic impedance $Z_{t}$ of the structure is obtained, and the normal incidence sound absorption coefficient can be obtained by substituting the sound absorption coefficient calculation formula

(15) $$\alpha =\frac{4Re\left(Z_{t}\right)}{{\left[1+Re\left(Z_{t}\right)\right]}^{2}+{\left[Im\left(Z_{t}\right)\right]}^{2}}$$

Under the condition of the same height of 20 mm, the sound absorption performances of the multi-layer microperforated plate structure and the traditional multi-layer microperforated plate structure are compared. Except for different sections, the other parameters of the two are the same, as shown in Table 1.

**Figure 3:**
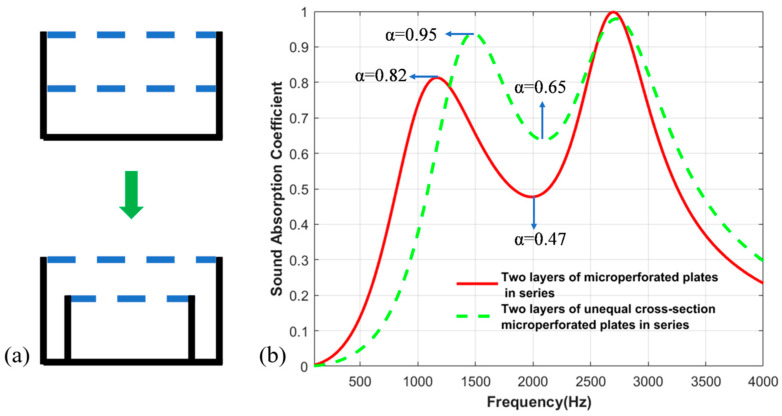
(**a**) Traditional double-layer structure and double-layer unequal section structure; (**b**) Two-layer equal-section microperforated plate series structure and two-layer unequal-section microperforated plate comparison of sound absorption coefficients.

Firstly, the double-layer microperforated plate structure with different cross-sections is compared with the traditional double-layer microperforated plate structure, and the results are shown in Figure 3. Obviously, the sound absorption performance of the double-layer microperforated plate structure is improved by the setting of unequal cross-sections; the first peak value is increased from 0.82 to 0.95, and the sound absorption trough is increased from 0.47 to 0.65.

**Figure 4:**
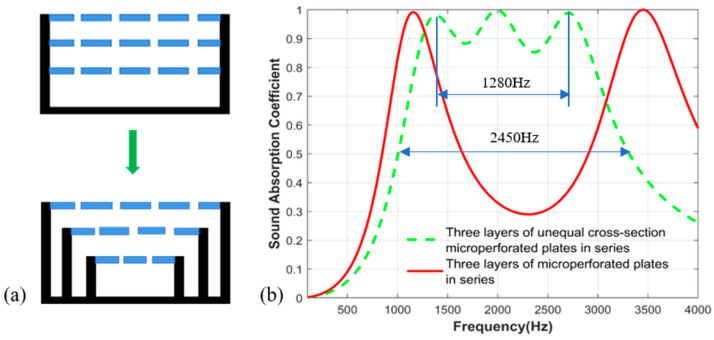
(**a**) Traditional three-layer structure and three-layer unequal section structure; (**b**) Three-layer equal-section microperforated plate series structure and three-layer unequal-section microperforated plate comparison of sound absorption coefficients.

Secondly, the three-layer microperforated plate structure with different sections is compared with the traditional three-layer microperforated plate structure, and the results are shown in Figure 4. The sound absorption coefficient of the structure with different cross-sections is relatively stable in a certain frequency range, the sound absorption performance is greatly improved, and the broadband sound absorption effect is realized.

## 3. Parametric Study

### 3.1. Effect of Perforation Ratio

**Figure 5:**
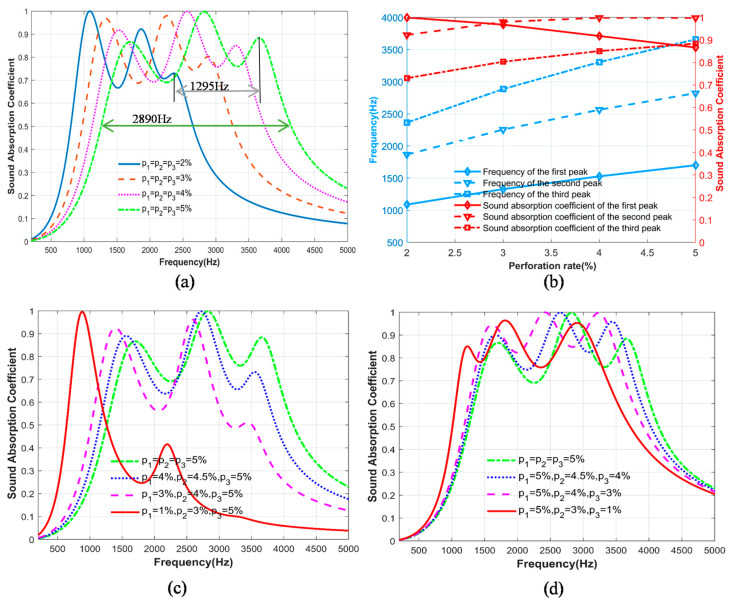
The effect of perforation ratio change on sound absorption performance: (**a**) Comparison of sound absorption coefficients under different perforation ratios; (**b**) Effect of perforation ratio on peak frequency and its absorption coefficient; (**c**) Comparison of sound absorption coefficients with increasing perforation ratio layer by layer; (**d**) Comparison of sound absorption coefficients with decreasing perforation ratio layer by layer.

The influence of the parameters of the series structure of the three-layer unequal-cross-section microperforated plate designed in this paper is studied and analyzed. To ensure that the cross-sectional area and the depth of the back cavity of each layer of the microperforated plate remain unchanged, the parameters are shown in Table 2. The perforation ratio of the three-layer microperforated plate is the same, and the perforation ratio of the three-layer microperforated plate changes at the same time. The sound absorption coefficient changes, within the frequency range of 800 Hz to 4150 Hz, by adjusting the perforation ratio, as shown in Figure 5a. The widest bandwidth achievable with variation in perforation rate is 2890 Hz. As the perforation ratio increases from p=2% to p=5%, the formant shifts to high frequency. As shown in Figure 5a, the maximum shift of the formant is 1295 Hz.

The influence of the change of perforation ratio on the resonance frequency of the structure and the sound absorption coefficient under the resonance frequency is shown in Figure 5b. The resonance frequencies of the first peak, the second peak and the third peak move to high frequency with the increase in perforation ratio, and the sound absorption coefficient under the resonance frequencies of the second and third peak also increases with the increase in perforation ratio. However, the sound absorption coefficient at the first peak resonance frequency decreases with the increase in the perforation ratio.

The above content regards the three-layer microperforated plate using the same perforation ratio. The perforation ratio changes the structure of the sound absorption coefficient of the impact of the three layers of the same perforation ratio; as can be seen from Figure 5a, the formants and valleys of the larger fall, the sound absorption coefficient in a certain frequency range is more unstable, and the sound absorption coefficient stays in a certain frequency range. The effect of this on the sound absorption coefficient of the structure was explored using different perforation rates of the three-layer microperforated plate, as shown in Figure 5c. From the first layer of microperforated panels to the third layer of microperforated panels, when the perforation ratio increases layer by layer, the sound absorption effect is poor and the sound absorption frequency band is narrow. As shown in Figure 5d, a structure with decreasing perforation ratios layer by layer is adopted. When the decrease is 1%—that is, the perforation ratios are $p_{1}=5\%$, $p_{2}=4\%$, and $p_{3}=3\%$—the structure can achieve a good sound absorption effect. The difference between the formants and valleys is small, and the minimum sound absorption coefficient of the valleys is 0.82, achieving broadband sound absorption.

### 3.2. Effect of Cross-Sectional Area

**Figure 6:**
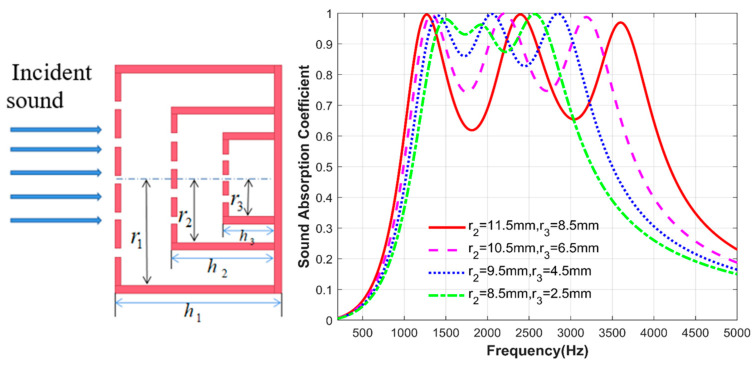
Comparison of sound absorption coefficients of the second and third layers of microperforated plates with different cross-sectional areas.

After ensuring that the perforation ratio and back-cavity depth of each layer of the microperforated plate remain unchanged, and other fixed parameters are shown in Table 3, the cross-sectional area of the second and third layers of the microperforated plate is changed to investigate the effect of cross-sectional area on the structural sound absorption coefficient. The radius of the first layer of the microperforated plate is fixed at 14.5 mm, as shown in Figure 6; with the change of the radius of the second and third layer of the microperforated plate, i.e., the change of the cross-sectional area, the first peak is basically unchanged, and when the radius of the two layers of the microperforated plate decreases, the second and third resonance frequencies are gradually shifted to the low frequency. At the same time, the acoustic absorption coefficients corresponding to the troughs of the wave are also gradually increased. When $r_{1}=14.5\;mm$, $r_{2}=8.5\;mm$, $r_{3}=2.5\;mm$, the minimum sound absorption coefficient corresponding to the wave valley is 0.88; at this time, the structure in a certain frequency range has a better sound absorption effect.

### 3.3. Effect of Back-Cavity Depth

**Figure 7:**
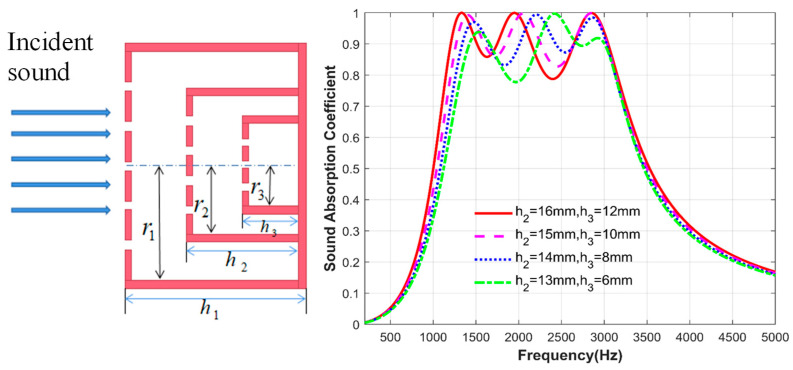
Comparison of sound absorption coefficients of the second and third layers of microperforated plates at different back-cavity depths.

Finally, the influence of the depth change of the back cavity of the second layer of the microperforated plate and the third layer of the microperforated plate inside the structure is studied. Similarly, other parameters of the structure are kept unchanged. The fixed parameters are shown in Table 4. Only the depth of the back cavity of the second and third layers of the microperforated plates is changed. As shown in Figure 7, the first and third peaks basically do not change with the depth of the back cavity. The main change is reflected in the second peak. As the depth of the back cavity of the two layer microperforated plates gradually decreases, the second resonance frequency gradually shifts to high frequency. Of course, it can be seen from the figure that the change in the depth of the back cavity brings less change in the sound absorption effect of the structure. This is because the overall structure is an ultra-thin sound absorber with a thickness of 20 mm, and the change in the depth of the internal back cavity causes a small change in the structural impedance.

## 4. Simulation Analysis

**Figure 8:**
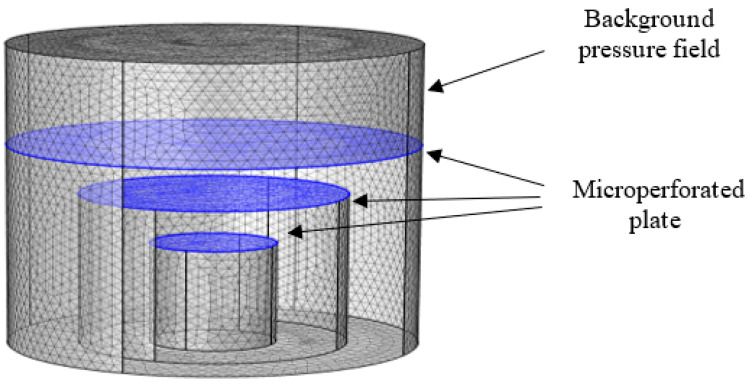
Finite element meshing.

In this paper, the pressure acoustic (frequency domain) module of COMSOL Multiphysics FE software is used to simulate and analyze the sound absorption performance of the series sound absorption structure model of unequal-cross-section microperforated plates. The simulation model mainly consists of a background pressure field, a built-in microperforated plate boundary, and a hard acoustic field boundary constructed using a tetrahedral mesh. The acoustic energy dissipation in the microperforated plate is described using the microperforated plate impedance boundary condition, and all boundary conditions are hard sound field boundary conditions (fully reflective, ignoring structural vibration, and considering only pressure acoustics), except for the microperforated plate boundary. The parameters used at this time are shown in Table 5. The outer wall of the structure is set as a rigid surface, and the air domain model after removing the outer wall is meshed by finite element, as shown in Figure 8.

**Figure 9:**
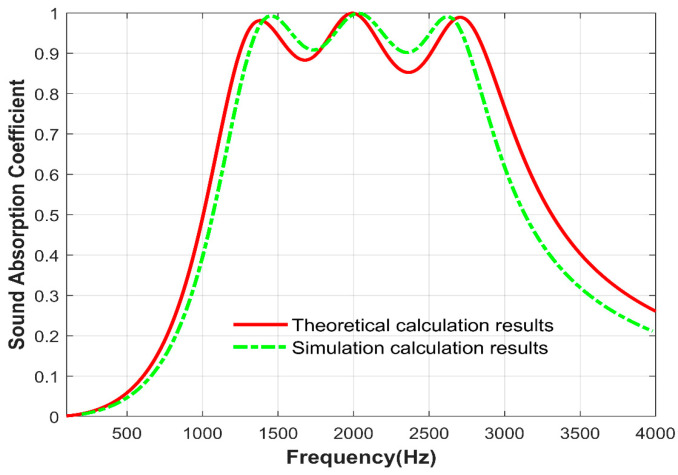
Comparison of simulation calculation results and theoretical calculation results.

The structural sound absorption coefficient variation with frequency, as shown in Figure 9, is compared with the theoretical calculation results. The variation in the sound absorption coefficient is approximately the same, and the different calculation methods within the simulation software lead to some errors.

**Figure 10:**
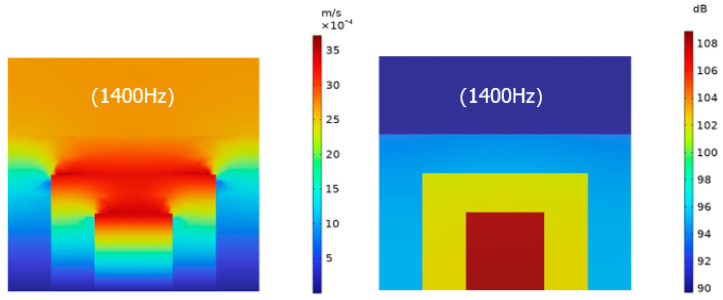
Sound velocity diagram and sound pressure level diagram at the first resonant frequency of 1400 Hz.

**Figure 11:**
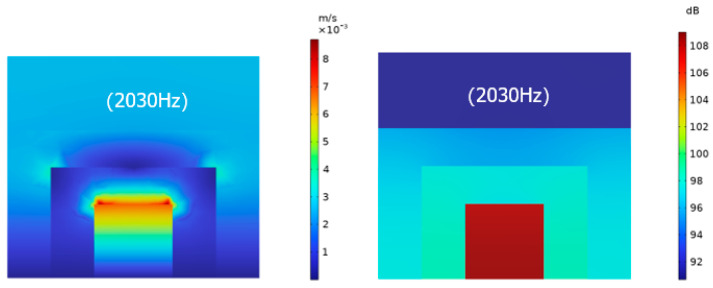
Sound velocity diagram and sound pressure level diagram at the second resonant frequency of 2030 Hz.

In order to further explore the mechanism of sound absorption of the structure, the sound pressure level and sound velocity diagrams of the structure at two resonance frequencies, as shown in Figure 10 and Figure 11, show that the sound pressure increases from the first layer of the microperforated plate to the third layer of the microperforated plate, and the sound pressure reaches the maximum in the third layer of the back cavity, and due to the change of the cross-section of each layer of the microperforated plate as well as the role of the micro perforation, it is higher in the vicinity of the microperforated plate. 

The sound speed drives the air particles to produce intense friction with the microperforated plate, which converts the sound energy into heat energy, thus resulting in the consumption of sound energy.

## 5. Experimental Analysis

**Figure 12:**
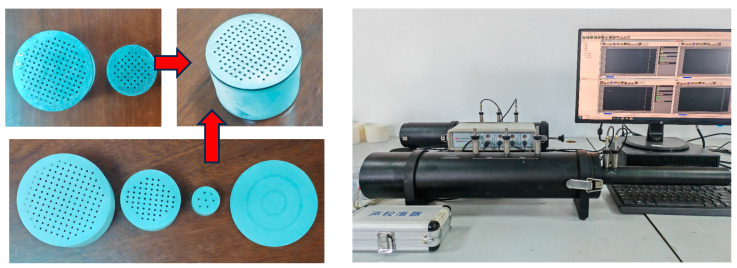
Impedance tube measurement system and 3D-printed experimental samples.

In this section, experiments to measure the absorption coefficient under normal incidence are carried out to verify the feasibility of the design method. According to ISO standard 10534-2 [37], the sound absorption coefficient of the entire structure is measured using an impedance tube measurement system and the standard dual-channel transfer function method. The measurement system mainly consists of speakers, power amplifiers, microphones and signal analyzers, as shown in Figure 12. Since the main sound absorption frequencies of the two structures are within the measurement range (500–6400 Hz) of the impedance tube with a diameter of 29 mm, the entire structure is fabricated as a 29 mm cylinder. The manufactured sample is shown in Figure 12. The microperforated plate is manufactured by laser perforation, and its back cavity is manufactured by photosensitive resin 3-D printing. The overall structure exhibits excellent mechanical stiffness and strength.

**Figure 13:**
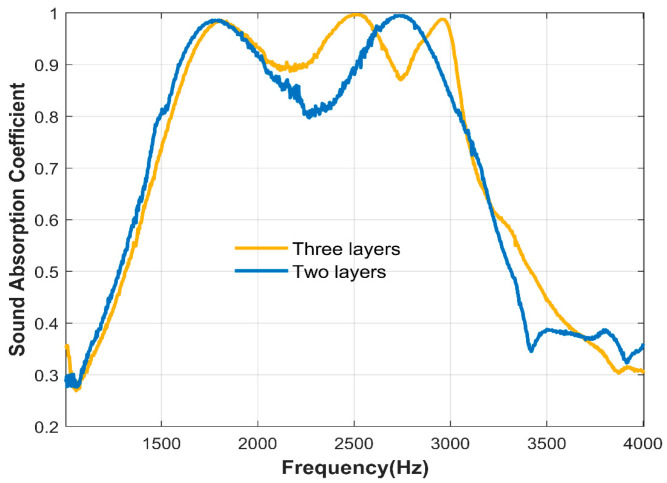
The sound absorption coefficients of two layers and three layers of unequal-cross-section microperforated plates in series were measured experimentally.

The two-layer unequal-cross-section microperforated plate series structure and the three-layer unequal-cross-section microperforated plate series structure are experimentally compared. The structural parameters are shown in Table 5. From the sound absorption coefficient diagram shown in Figure 13, it can be seen that the three-layer structure has three resonance peaks, and the two-layer structure has two resonance peaks; that is, the microperforated plate of each layer provides a resonance peak.

**Figure 14:**
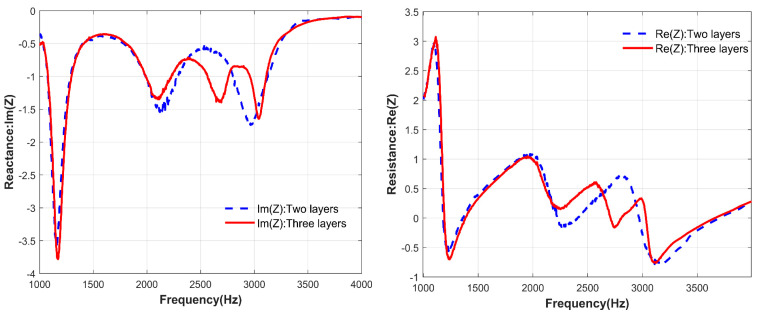
The acoustic resistance and acoustic impedance changes of the series structure of two-layer and three-layer unequal-cross-section microperforated plates were measured experimentally.

Figure 14 shows the contrast of acoustic resistance and acoustic reactance of the two-layer structure and three-layer structure. According to Formula (15), when the real part of the acoustic impedance is close to equal to 1 and the imaginary part is close to equal to 0, the acoustic impedance matching conditions are met, and the structure achieves nearly perfect sound absorption. As shown in Figure 14, from 1000–4000 Hz, the acoustic reactance of the double-layer structure has two peaks close to 0, and the acoustic resistance has two peaks close to 1, corresponding to the two absorption peaks of the double-layer structure. Similarly, when a layer of series connection is added to the structure, the acoustic impedance changes. Near the resonance frequency, the acoustic resistance and acoustic reactance of the three-layer structure have one more wave peak than those of the two-layer structure, corresponding to the three absorption peaks of the three-layer structure. It is proved that under the same thickness, increasing the number of structural layers can improve the sound absorption effect of the structure.

**Figure 15:**
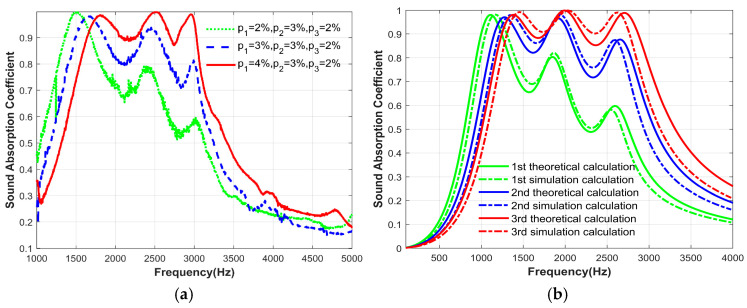
The change in sound absorption coefficient of the structure as the perforation ratio of the first layer of microperforated plate changes: (**a**) Experimentally measured; (**b**) Theoretical calculation results and simulation calculation results.

**Figure 16:**
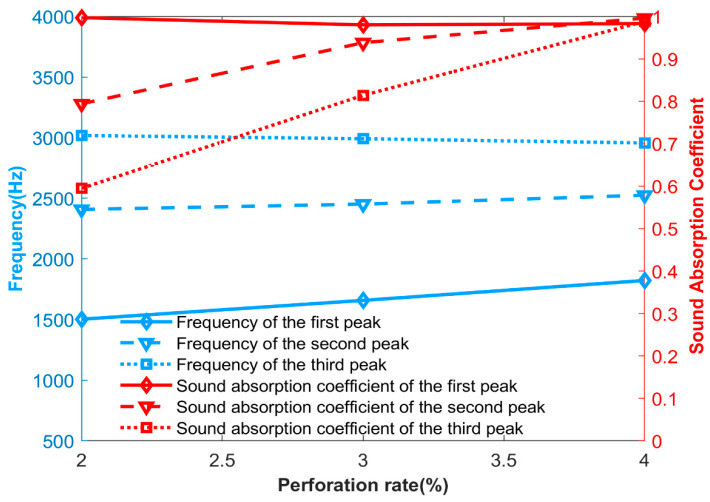
The relationship diagram between the formant, sound absorption coefficient and the perforation rate of the first layer of microperforated plates, measured by experiment.

Only the perforation ratio of the first layer of the microperforated plate was varied, while other structural parameters remained unchanged. Other parameters are shown in Table 6, and the parameters of perforation ratio are shown in Table 7. The experimental data were also compared with theoretical and simulation data to verify the feasibility. Figure 15 shows the sound absorption coefficients of experimental, theoretical and simulation, respectively. As the perforation ratio of the first layer of the microperforated plate changes, the variation trend of the two sound absorption coefficients is similar, verifying the reliability of the theory. Figure 16 shows the relationship between the resonance frequency, sound absorption coefficient, and the change in the perforation ratio of the first layer of the microperforated plates obtained from the experiment. Combined with the sound absorption coefficient diagram and the relationship diagram, the change in perforation ratio of the first layer of the microperforated plates mainly affects the sound absorption coefficients at the second and third resonance frequencies. It can also be concluded that the third resonance peak of the structure is caused by the first layer of the microperforated plates.

## 6. Conclusions

In conclusion, this paper investigates a microperforated plate acoustic structure with unequal cross-sections in series, which increases the acoustic velocity and thus the acoustic energy loss through the mutation of the cross-section of each layer. Compared with the conventional microperforated plate series structure, the structure designed in this paper can pull up the peaks and valleys at the same thickness, which improves the overall acoustic performance. This simple structure allows for light and thin broadband sound absorption. Unequal cross-section microperforated panels with a total thickness of 20 mm and a diameter of 29 mm provide good absorption in the frequency range 1000-3500; the absorption coefficients reach 1 for all three peaks and as low as 0.9 for the troughs, which greatly improves the absorption coefficient compared to the traditional series connection. 

In this paper, the effects of parameters such as perforation ratio, cross-section, and depth of the back cavity on the structure are investigated, and the feasibility of the structure is verified by theoretical, simulation, and experimental analyses, which provides more ideas for the study of broadband sound absorption.

## Figures and Tables

**Table 1:** Parameters of three-layer unequal-cross-section microperforated plate series structure and conventional series structure.

	Aperture (mm)	Perforation Ratio (%)	Cross-Section Radius (mm)	Back-Cavity Depth (mm)
First layer	0.5	4	14.5	20
Second layer	0.5	3	9.5	15
Third layer	0.5	2	4.5	10

**Table 2:** Fixed parameters of the structure in addition to the perforation ratio.

	Aperture (mm)	Cross-Section Radius (mm)	Back-Cavity Depth (mm)
First layer	0.5	14.5	20
Second layer	0.5	9.5	15
Third layer	0.5	4.5	10

**Table 3:** Fixed parameters of the structure in addition to the cross-section radius.

	Aperture (mm)	Perforation Ratio (%)	Back-Cavity Depth (mm)
First layer	0.5	4	20
Second layer	0.5	3	15
Third layer	0.5	2	10

**Table 4:** Fixed parameters of the structure in addition to the back-cavity depth.

	Aperture (mm)	Perforation Rate (%)	Cross-Section Radius (mm)
First layer	0.5	4	14.5
Second layer	0.5	3	9.5
Third layer	0.5	2	4.5

**Table 5:** Parameters of the overall structure.

	Aperture (mm)	Perforation Ratio (%)	Cross-Section Radius (mm)	Back-Cavity Depth (mm)
First layer	0.5	4	14.5	20
Second layer	0.5	3	9.5	15
Third layer	0.5	2	4.5	10

**Table 6:** Fixed parameters of the structure in addition to the perforation ratio.

	Aperture (mm)	Cross-Section Radius (mm)	Back-Cavity Depth (mm)
First layer	0.5	14.5	20
Second layer	0.5	9.5	15
Third layer	0.5	4.5	10

**Table 7:** Perforation ratios of different states.

	1st	2nd	3rd
Perforation ratio of the first layer *p*_1_ (%)	2	3	4
Perforation ratio of the second layer *p*_2_ (%)	3	3	3
Perforation ratio of the second layer *p*_3_ (%)	2	2	2

## Data Availability

The data supporting the findings in this article is available upon reasonable request from the corresponding author due to privacy.
